# Assessing Adherence to Antihypertensive Medication by Means of Dose-Dependent Reference Plasma Concentration Ranges and Ultra-High Performance Liquid Chromatography-Ion Trap Mass Spectrometry Analysis

**DOI:** 10.3390/molecules26051495

**Published:** 2021-03-09

**Authors:** Lea Wagmann, Aline C. Vollmer, Lucas Lauder, Felix Mahfoud, Markus R. Meyer

**Affiliations:** 1 Center for Molecular Signaling (PZMS), Institute of Experimental and Clinical Pharmacology and Toxicology, Department of Experimental and Clinical Toxicology, Saarland University, 66421 Homburg, Germany; lea.wagmann@uks.eu (L.W.); aline.vollmer@uks.eu (A.C.V.); 2 Klinik für Innere Medizin III, Kardiologie, Angiologie und Internistische Intensivmedizin, Universitätsklinikum des Saarlandes, Saarland University, 66421 Homburg, Germany; lucas.lauder@uks.eu (L.L.); felix.mahfoud@uks.eu (F.M.); 3 Institute for Medical Engineering and Science, MIT, Cambridge, MA 02142, USA

**Keywords:** hypertension, antihypertensive drugs, bioanalysis, adherence monitoring, LC-MS/MS

## Abstract

Poor adherence to antihypertensive drug therapy is a well-recognized problem and can be assessed by mass spectrometry-based analyses of body fluids. However, contrary statements exist whether drug quantification in blood or qualitative screening in urine is more suitable. The present pilot study aimed to further elucidate the power of blood plasma drug concentrations for adherence monitoring by developing and validating a quantification procedure for nine antihypertensive drugs (amlodipine, bisoprolol, candesartan, canrenone, carvedilol, metoprolol, olmesartan, torasemide, and valsartan) in blood plasma using liquid–liquid extraction and an ultra-high-performance liquid chromatography-ion trap mass spectrometry analysis. The procedure should then be used for an adherence assessment and compared with the results of an established qualitative urine screening. Selectivity, carryover, matrix effect, accuracy, precision, dilution integrity, and stability were successfully validated, except for amlodipine. The applicability was demonstrated by analyzing 19 plasma samples containing 28 antihypertensive drugs and comparing the measured concentrations with calculated dose-dependent reference plasma concentration ranges. The interpretation of plasma concentrations was found to be more sophisticated and time-consuming than that of urine screening results, and adherence could not be assessed in two cases (10%) due to measured plasma concentrations below the lower limit of quantification. However, 14 out of 19 subjects were classified as adherent (75%) and three as nonadherent (15%), in contrast to 19 (100%) that were claimed to be adherent based on the results of the qualitative urine screening. Nevertheless, further data is needed to estimate whether plasma quantification is superior in terms of assessing adherence to antihypertensive medication.

## 1. Introduction

Hypertension remains one of the most prevalent cardiovascular risk factors leading to more than nine million premature deaths each year [1]. Blood pressure lowering has been shown to reduce the risk of major cardiovascular disease events, such as stroke, heart failure, coronary heart disease, and all-cause mortality [2,3]. The current guidelines recommend the administration of antihypertensive drugs such as beta-blockers, calcium antagonists, diuretics, angiotensin converting enzyme inhibitors, or angiotensin receptor blockers (ARBs) [3,4], preferably in fixed-dose combinations.

According to the WHO, adherence is “the extent to which a person’s behavior […] corresponds with agreed recommendations from a health care provider”, and adherence to prescribed medication poses an essential part [5]. Poor adherence to antihypertensive medication is frequently observed in patients with uncontrolled blood pressure and might be an underlying cause for hypertensive urgencies [6,7,8] but can also be mistaken for treatment resistance [9]. Numerous studies have reported that about half of hypertensive patients do not take their blood pressure-lowering medications as prescribed [9,10,11,12]. Hence, the assessment of patients’ adherence to antihypertensive medication is becoming an increasingly recognized component of successful hypertension management [5]. These measures include interviews, diaries, or questionnaires that are easily accessible, fast, and inexpensive but were shown to overestimate the adherence [13,14]. The counting of remaining dosage units such as tablets or the collection of refill data represent objective strategies but have limited reliability [6]. Electronic monitoring devices, so-called medication event monitoring systems that record the time and date when a medication container was opened, are a recent innovation in this field, but the expense of these devices precludes their widespread use and opening them does not necessarily prove any intake [5]. Bioanalytical methods, which may be considered as the gold standard [6], allow the unambiguous detection of drugs and/or their metabolites in body fluids and, thus, a proof an intake. Hence, they directly and objectively assess patients’ adherence. Such methods are of increasing importance due to the high specificity and sensitivity of, particularly, liquid chromatography (LC) coupled to low-resolution or high-resolution mass spectrometry (HRMS) [6,9,15,16,17]. Furthermore, repeated measurements were found to improve the adherence [18].

Amongst the analyzed body fluids, urine has become the matrix of choice for qualitative screening of antihypertensive drugs [9,17,19], but blood plasma [20] and oral fluid [15] were described to also be suitable. Almost all drugs or their metabolites can be detected in urine, and their complete absence guarantees that the medication has not been taken for a duration equivalent to several half-lives [6]. Urine can be obtained noninvasively in large volumes, and the drugs, as well as their metabolites, are usually concentrated [21]. Nevertheless, some major disadvantages of urine screening approaches were described, such as the elaborate sample collection, simple adulteration, and misclassification of drug adherence [22,23]. Compounds with a short half-life and/or low assay sensitivity could yield false negative classifications, while compounds with a long half-life and/or high assay sensitivity could yield false positive classifications for adherence [22]. Thus, recent data indicated that a thorough analysis of the blood serum or plasma may be beneficial over urine [22,24,25,26,27]. Accompanying the blood pressure measurement creates the opportunity to link the drug concentration to the blood pressure profile [22].

The present pilot study aimed to further elucidate the power of measuring blood plasma drug concentrations for adherence monitoring. Therefore, several objectives were pursued within the framework of the current study. First, to develop and validate an adherence monitoring method based on plasma quantification by ultra-high-performance (UHP) LC-ion trap mass spectrometry (ITMS). The method should cover frequently prescribed antihypertensive drugs [28,29] and be developed and validated in accordance with guidelines of the European Medicines Agency (EMA) [30]. Second, to apply this method to assess adherence by using dose-dependent reference plasma concentration ranges. As the findings should finally be compared with those of an established, metabolite-based urine screening procedure by LC-HRMS [17], only plasma samples with matched urine samples that tested positive for antihypertensive drugs should be included in the applicability testing to enable a comparison of the adherence.

## 2. Results

### 2.1. Development of the Analytical Procedure

**Figure 1:**
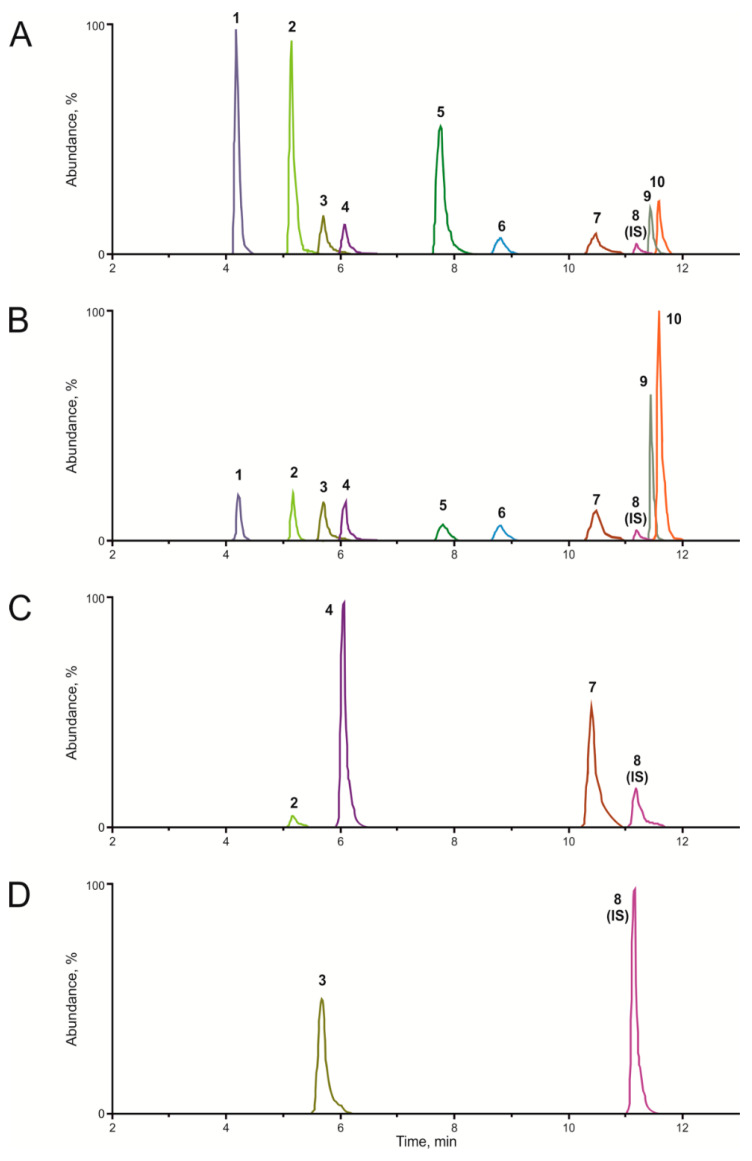
Reconstructed ion chromatograms of the *m/z* of the antihypertensive drugs and the internal standard (IS) diazepam-d5 (1 metoprolol, 2 bisoprolol, 3 olmesartan, 4 torasemide, 5 carvedilol, 6 amlodipine, 7 candesartan, 8 IS diazepam-d5, 9 valsartan, and 10 canrenone). (**A**) Methanolic solution, antihypertensive drugs 1 mg/L each, IS 0.1 mg/L. (**B**) Extracted high-quality control sample. (**C**) Extracted plasma sample after intake of bisoprolol, torasemide, and candesartan. (**D**) Extracted plasma sample after intake of olmesartan.

Nine antihypertensive drugs with different chemical properties were chosen for the current pilot study, including the beta-blockers bisoprolol, carvedilol, and metoprolol; the diuretics canrenone and torasemide; the ARBs candesartan, olmesartan, and valsartan; and the calcium antagonist amlodipine. A representative and reconstructed ion chromatogram of the *m/z* of the antihypertensive drugs (1 mg/L, each) and the internal standard (IS) diazepam-d5 (0.1 mg/L) is depicted in Figure 1A. Different signal intensities despite the same concentration were observed, most probably due to differences in the ionization efficiency.

Liquid–liquid extraction (LLE) was found as suitable sample preparation procedure. However, the addition of formic acid was crucial for the extraction of the acidic antihypertensive drugs candesartan, olmesartan, and valsartan from blood plasma. Repeated vortexing and shaking was needed to reliably homogenize the samples during sample preparation. Figure 1B shows a plasma extract at a high level of quality control (QC) (for concentrations, see Table 1) with an IS concentration of 10 ng/mL. The sample preparation procedure was tested for interferences preventing the reducibility by determination of the coefficients of variation (CVs) of the peak area ratios after three extractions. CVs were found to be between 2% (torsemide) and 12% (metoprolol) and, thus, acceptable. All analytes were successfully separated via UHPLC within 12 min, obtaining retention times (t_R_) between 4.2 and 11.7 min (details are given in Table 2). The total run time was 16 min per run. Furthermore, the smartMRM mode increased the UHPLC-ITMS sensitivity compared to autoMS^n^ mode, which is used by default for plasma screening and quantification aiming to detect intoxications in emergency toxicology [31].

### 2.2. Method Validation

Atorvastatin-d5 and atorvastatin, as well as diazepam-d5 and diazepam, were found to coelute and were therefore tested for the effects of the nondeuterated compounds on the peak area of the deuterated compounds. Ion suppression of atorvastation-d5 (−17%) was detected in the presence of atorvastatin, while diazepam did not influence the diazepam-d5 peak area. Therefore, further analyses were performed using only diazepam-d5 as IS.

Method validation was performed in accordance with the “Guideline on bioanalytical method validation” of the EMA [30]. Selectivity was given for all antihypertensive drugs, as well as for the IS diazepam-d5. No carryover was observed, except for torasemide with an analyte response greater than 20% of its lower limit of quantification (LLOQ), which corresponded to the concentration of Cal 1. However, the analyte response was not greater than 20% of Cal 2. After five washing runs injecting an eluent mixture A/B (1:1, *v/v*, see Materials and Methods) immediately after the analysis of Cal 6, the analyte response was below 20% of the LLOQ of torasemide.

A linear calibration model was successfully applied using a six-point calibration of all analytes. However, different weighing factors (equal, 1/x, 1/x^2^) had to be used. The details are given in Table 1. The acceptance criteria (AC) were checked for each individual calibration. Calibration parameters such as slope, y-axis intercept, and the deviation of the back-calculated concentrations of the three calibrations acquired during the validation procedure are available in the Supplementary Materials (Table S1). If the AC for the back-calculated concentrations were not fulfilled, this calibration standard sample was rejected for the specific analyte, and the calibration without this calibration standard was reevaluated, including a regression analysis according to the guidelines [30]. Nevertheless, at least five out of six calibration standards fulfilled these criteria for each calibration, and neither Cal 1 nor Cal 6 had to be excluded in accordance with the EMA guidelines.

Concerning the matrix effect experiments, Table 3 summarizes the IS-normalized matrix factors (MF) of all analytes, ranging from 0.82 (candesartan) to 2.56 (amlodipine) for the low QC and 0.96 (candesartan and olmesartan) to 1.37 (amlodipine) for the high QC. CVs were not greater than 15% for bisoprolol, canrenone, and torasemide in the case of low QC, while all other analytes exceeded the AC with CVs between 17% (metoprolol) and 42% (valsartan). For high QC, CVs were not greater than 15% for all analytes except amlodipine (16%), candesartan, olmesartan (both 17%), and valsartan (25%). In general, the matrix effects were observed to be more pronounced in the case of low analyte concentrations, but the difference between the IS-normalized MF determined for low and high QC was highest in the case of amlodipine.

The accuracy of an analytical method describes the closeness of the determined value obtained by the method to the nominal concentration of the analyte. The precision of the analytical method describes the closeness of repeated individual measures of the analyte [30]. The within-run accuracy was found to be acceptable with mean concentrations within ±15% of the nominal values for the low, medium, and high QC samples (1–15%) and within ±20% for the LLOQ QC samples (2–15%) for all analytes, except for amlodipine (medium QC, 17%), carvedilol (medium QC, 21%), torasemide (LLOQ QC, 34% and low QC, 20%), and valsartan (low QC, 20%). The between-run accuracy was found to be acceptable, with mean concentrations within ±15% of the nominal values for the low, medium, and high QC samples (0–15%) and within ±20% for the LLOQ QC samples (1–19%) for all analytes, except for carvedilol (LLOQ QC, 28%), olmesartan (LLOQ QC, 26%), and torasemide (low QC, 26%). The within-run and between-run precision were expressed as the CV and found to be acceptable with CV values within 15% for the low, medium, and high QC samples (1–14%) and within 20% for the LLOQ QC samples (3–20%) for all analytes with the exception of valsartan (within-run precision, medium QC, CV 19%).

After the dilution of samples spiked above the ULOQ (= Cal 6), the determined concentrations were found to be precise (CVs of 6–15%) for all analytes. The determined values were also found to be accurate for bisoprolol, candesartan, canrenone, olmesartan, and valsartan (1–12% variation). In the case of amlodipine (25%), carvedilol, metoprolol (both 30%), and torasemide (17%), the AC of the EMA concerning accuracy after dilution were exceeded.

Methanolic stock solutions were found to be stable at −20 °C with CVs below 15% for all analytes. All analytes were found to be stable in plasma extracts after 20 h in the autosampler at 8 °C, except for amlodipine with a decrease of 16% compared to the nominal concentration.

### 2.3. Applicability and Assessment of Adherence by Using the Dose-Dependent Reference Plasma Concentration Range

Applicability testing was performed using 19 different plasma samples. A total of 28 antihypertensive drugs were prescribed (7 × bisoprolol, 5 × candesartan, 1 × canrenone, 1 × carvedilol, 2 × metoprolol, 3 × olmesartan, 6 × torasemide, and 3 × valsartan), and all of them could successfully be identified in plasma. Three different antihypertensive drugs were present in two samples (ID 6 and 14) and two drugs in five samples (IDs 4, 11, 12, 13, and 18), while one drug was quantified in the remaining 12 plasma samples (IDs 1, 2, 3, 5, 7, 8, 9, 10, 15, 16, 17, and 19). Reconstructed ion chromatograms after the analysis of two samples are given in Figure 1C (sample ID 14) and Figure 1D (sample ID 10). Previously, matching urine samples were tested positive for the antihypertensive drugs and/or their metabolites using an established, metabolite-based urine screening procedure by LC-HRMS [17].

The plasma quantification results given in Table 4 also contained the therapeutic plasma concentration range described in the literature [32]; they prescribed daily doses of the antihypertensive drugs and calculated dose-dependent reference plasma concentration ranges with the underlaying pharmacokinetic parameters taken from Baselt [33]. The calculated dose-dependent reference plasma concentration ranges were defined to comprise c_min–20%_ to c_max_. The trough plasma concentration 24 h after the latest drug intake, c_min_, was calculated using the elimination half-life t_1/2_, peak plasma concentration c_max_, and the corresponding time after intake when c_max_ was reached (t_max_) for the corresponding dose described by Baselt [33]. The shortest given elimination half-life t_1/2_ was always used for the calculation. A subtraction of 20% from c_min_ led to c_min–20%_, which was defined as lower end of the calculated dose-dependent reference plasma concentration range. The peak plasma concentration c_max_ was defined as the upper end. A summary of the pharmacokinetic parameters used for calculation of the reference plasma concentration ranges, as well as an example for such a calculation, are available as Supplementary Materials Tables S2 and S3, respectively.

The dose used for the calculation of the reference plasma concentration range was given in brackets if it was different from the patient’s prescribed daily dose. The measured plasma concentration was above the ULOQ of olmesartan, torasemide, or valsartan, respectively, in the case of six samples (IDs 3, 6, 8, 11, 14, and 19), which were diluted (1:10 with pooled blank plasma), extracted, and reanalyzed. The measured plasma concentration was still above the ULOQ of valsartan in the case of one sample (IDs 11), and it was thus diluted (1:20 with pooled blank plasma), extracted, and reanalyzed.

Compared to the therapeutic plasma concentration ranges described in the literature [32], the determined plasma concentrations of 12 out of 28 antihypertensive drugs were within (43%), 15 below (53%), and one above (4%) the given ranges. Compared to the calculated dose-dependent reference plasma concentration ranges, the determined plasma concentrations of 21 out of 28 antihypertensive drugs were within (75%), three below (11%), and two above (7%) the range. In the case of two antihypertensive drugs, the determined plasma concentrations were below the LLOQ (7%). However, the latter was higher than c_min–20%_ in both cases.

## 3. Discussion

### 3.1. Development and Validation of the Analytical Procedure

The chosen antihypertensive drugs, including beta-blockers, ARBs, diuretics, and a calcium antagonist, are frequently prescribed [28]. Canrenone can be used as diuretic drug itself but also represents the active main metabolite of its prodrug spironolactone [34]. The method is therefore suitable for the adherence monitoring of both drugs. All of these antihypertensive drugs are also available in fixed-dose combinations, the use of which is recommended by the current guidelines [28].

Only 100 µL of blood plasma were required for the analysis. The duration of a single analytical run (including equilibration) of 16 min is expected to be appropriate and applicable in the daily laboratory routine. A single injection and analysis per sample saved run time and was found to be adequate during the method development. Diazepam-d5 was chosen as a suitable IS. In contrast to atorvastatin-d5, which provided a higher t_R_ (12.4 min) than all the antihypertensive drugs, diazepam-d5 eluted between candesartan and valsartan (see Table 2). Furthermore, neither ion suppression nor the enhancement of its peak area was detected in the presence of coeluting diazepam. Remane et al. [35] demonstrated the importance of ion suppression and enhancement experiments for developing LC-based mass spectrometry procedures if the baseline separation is not possible, and the current results confirmed their findings. Some patients may be advised to take atorvastatin, diazepam, and antihypertensive drugs at the same time. Ion suppression and enhancement of the peak area of the IS may lead to false quantification results. Atorvastatin suppressed the atorvastatin-d5 peak area, which may lead to overestimation of the antihypertensive drug plasma concentrations. In contrast, diazepam did not influence the diazepam-d5 area, and the current method is therefore also applicable for patients advised to take diazepam. No other analytes were tested for ion suppression or enhancement due to sufficient chromatographic separation (see Figure 1A).

The bioanalytical methods applied for measuring drug concentrations in biological matrices have to be validated in order to provide reliable results [30]. The developed method was fully validated according to the EMA guidelines [30]. The limitations observed during validation will be discussed in the following. Due to the carryover of torasemide, several washing runs injecting the eluent mixture A/B (1:1, *v/v*, see Materials and Methods) are recommended after the analysis of Cal 6, high QC, or torasemide-containing patient samples to ensure that the analyte response is below 20% of the LLOQ. Based on the validation procedure, five washing runs were recommended. However, this option is quite time-consuming (>1 h). Washing runs are also recommended if patient samples containing plasma concentrations above those of Cal 6 were analyzed. In these cases, the washing run should be checked for carryover of the analyte. In the case of torasemide, the elevation of its LLOQ to the concentration of Cal 2 (25 ng/mL) represents a less time-consuming alternative, which may complicate the adherence assessment of low torasemide plasma concentrations. The first option, consisting of washing runs, was chosen for the subsequent validation and application experiments described in the current study, but the latter may be more suitable for routine application of the presented method.

The EMA guidelines do not define AC for the MF but for the CV of the IS-normalized MF calculated from six lots of matrix that should not exceed 15%. However, CVs were >15% in the case of six analytes for low QC and in the case of four analytes for high QC. However, half of them (two analytes for low QC and three analytes for high QC) had CVs between 15% and 20%. Other guidelines for the quantitative determination of drugs, such as the “Recommendations of criteria for development and validation of analytical methods for estimating concentrations of drugs in blood to be used in 24/7 clinical toxicology” published by the Society of Toxicological and Forensic Chemistry (GTFCh) [36], include different instructions for assessing matrix effects. The GTFCh recommendations stated that the matrix effect should not exceed ±30%. This means, for the current study, that IS-normalized MF between 0.70 and 1.30 would be acceptable. According to this recommendation, all analytes fulfilled the AC except for amlodipine in low and high QC.

According to the EMA guidelines, the mean concentration determined in the accuracy experiments should be within ±15% of the nominal value (LLOQ ±20%) [30]. The accuracy of the AC was not fulfilled for the individual concentration levels in the case of five antihypertensive drugs (amlodipine, carvedilol, olmesartan, torasemide, and valsartan). However, the AC were still within 25% in most cases. For precision experiments, CVs should not exceed 15% (LLOQ 20%), and the precision AC were only exceeded in the case of valsartan but still within 20%. In comparison, the aforementioned recommendations of the GTFCh allow 30% of the nominal value of two QC levels at the lower (20%) and upper (80%) end of the calibration range, as well as the precision of <30% [36]. Accordingly, the current validation experiments would be in line with those recommendations, except for torasemide concerning the within-run accuracy at the LLOQ QC level (34%) supporting the elevation of the LLOQ of torasemide to the concentration of Cal 2.

After dilution, the determined concentrations turned out to be precisely in accordance with the EMA AC, while the quantification results of four antihypertensive drugs exceeded the accuracy AC of the EMA. Nevertheless, the quantified concentrations did not exceed the cut-off of ±30% of the nominal value, which was recommended by the GTFCh.

According to the EMA guidelines, evaluation of the stability must be carried out to ensure that every step taken during the sample preparation and sample analysis, as well as the storage conditions used, do not affect the concentration of the analyte [30]. The current experiments investigated whether the storage conditions used for the spiking solutions, as well as the conditions in the autosampler during short-term storage of the plasma extracts, influenced the analytes’ concentrations. The stability of methanolic stock solutions could be ensured for the whole validation process and subsequent applicability testing. However, it should be regularly retested in case the current method is used for routine work. Extracted plasma samples were stable for at least 20 h in the autosampler. It is therefore recommended to finalize the batch analysis within this timeframe.

The stability of the analytes in the current sample matrix plasma was not investigated, as this information was available from the literature [33]. Baselt described all analytes to be stable for 24 h in plasma if stored at room temperature and for at least a month if stored in the freezer. This means that plasma should be separated by centrifugation immediately after sample collection. If samples cannot be transferred to the laboratory within 24 h, it is recommended to freeze them and ship them frozen to ensure the analytes’ stability.

In summary, the current method was successfully validated and fulfilled the AC of the EMA and/or the GTFCh [30,36]. It is therefore regarded as applicable for adherence monitoring of hypertensive patients by drug quantification in blood plasma. The only exception is amlodipine, whose IS-normalized MF exceeded the AC of both guidelines in low and high QC. Furthermore, amlodipine is known to be photosensitive [26], but all amlodipine-containing solutions were handled in amber-colored vials during method development and validation. The amlodipine plasma concentrations should be interpreted with caution, and clinicians must be advised to immediately protect amlodipine-containing plasma samples from light. As both high matrix effects and photosensitivity might impact a reliable quantification, amlodipine was not included in the applicability study.

### 3.2. Applicability and Assessment of Adherence by Using the Dose-Dependent Reference Plasma Concentration Range

Applicability was tested using 19 plasma samples containing 28 antihypertensive drugs. Each of the eight antihypertensive drugs that passed the validation procedure contained at least in one plasma sample (see Table 4). Interpretation of the analytical results is one crucial step. Classification of the plasma concentrations is more sophisticated and time-consuming than the evaluation of qualitative urine screening results, where the presence or absence of a drug and/or its metabolites represents the main criterion.

Thus, the measured plasma concentrations were compared to the plasma concentrations described as therapeutic (“normal”) in the paper by Schulz et al. [32]. These data are intended to help toxicologists to decide whether a patient may suffer from an intoxication and do not focus on adherence monitoring. Only a single range is given for each drug, independent of the prescribed daily dose. Plasma concentrations of only 12 antihypertensive drugs were found to be within the described therapeutic range, while even more were below it. These findings are in accordance with the observations by Ritscher et al. [25]. They developed a plasma quantification procedure including four beta-blockers (amongst them, bisoprolol and metoprolol) and four diuretics (amongst them, canrenone and torasemide) with a focus on the adherence assessment and compared their quantification results with the therapeutic plasma concentration ranges according to the article by Schulz et al. from 2012 [37]. About 30% were below the described therapeutic concentration ranges, and the authors concluded that these ranges cannot be used to evaluate adherence, as drug ingestion was monitored in their study. Therefore, Ritscher et al. used a dose-related concentration approach and compared measured concentrations with trough serum drug concentrations calculated individually for each patient using data from pharmacokinetic studies, including bioavailability, total body clearance, and elimination rate constants [25]. Rognstad et al. also addressed the problem of lacking serum reference ranges of antihypertensive drugs and preventing the widespread use of therapeutic drug monitoring in cardiology [38]. Nevertheless, the serum concentrations measured by Ritscher et al. [25] were comparable to the plasma concentrations determined in the current study. In the case of bisoprolol, Ritscher et al. described the trough serum levels between 8.9 and 41 ng/mL for patients with prescribed daily doses between 2.5 and 10 mg. Two trough serum concentrations each were described for canrenone (26 and 48 ng/mL, daily spironolactone dose 25 mg, each), as well as metoprolol (6.6 and 13 ng/mL, daily dose 50 or 100 mg, respectively). Concerning torasemide, Ritscher et al. reported trough serum levels between 18 and 370 ng/mL for patients with prescribed daily doses of 5 or 10 mg [25].

In the current study, drug intake was not monitored, and the exact time between intake and sampling was unknown, as is usually the case for outpatients. Therefore, adherence cannot be assumed, even if matching urine samples were tested positive for the antihypertensive drugs and/or their metabolites [17]. Instead, a simplified assessment approach was used by calculating the dose-dependent reference plasma concentration range (c_min–20%_–c_max_) for each antihypertensive drug by using data from Baselt [33], such as the peak plasma concentrations c_max_, the time at which c_max_ was reached (t_max_), and the elimination half-life t_1/2_. The latter usually represents a range, and the shortest given half-life was used for the calculations. The dosing regimens were simplified by calculating the total daily dose with a hypothetical dosing interval of 24 h according to Hiemke et al. [39]. The least-expected plasma concentration at 24 h after the latest drug intake (trough concentration) was c_min_. A subtraction of 20% led to c_min–20%_ which was defined as the lower end of the reference plasma concentration range to exclude the influence of interindividual differences and measurement uncertainties. As Baselt [33] did not report peak plasma concentrations for all doses prescribed to the patients, the quantification results were evaluated using the most comparable dose of which the peak plasma concentration c_max_ was available.

The determined plasma concentrations of 21 out of 28 antihypertensive drugs were found to be within the calculated dose-dependent reference plasma concentration ranges underlining the importance of including the individual daily dose and trough concentrations. Three plasma concentrations were below the calculated reference ranges, but the drugs could successfully be identified in plasma. In the case of canrenone (sample ID 4), the patient was advised to take only 50 mg per day, but the peak plasma concentration c_max_ was only available after an intake of 100 mg. In the case of olmesartan (sample ID 1 and 10), the concentrations were slightly below the lower limit of the reference plasma concentration ranges. In all three cases, a follow-up analysis may be recommended.

Two plasma concentrations were above the calculated reference concentration ranges. However, the upper limit of the reference range was defined by the peak plasma concentration c_max_. This value only reflects the mean value of a patient collective. In conclusion, higher peak plasma concentrations may be expected in some patients, leading to the conclusion that patients with plasma concentrations exceeding the given peak plasma concentration c_max_ (e.g., sample ID 3 and 12) are considered as adherent. Furthermore, the concentrations of olmesartan, torasemide, or valsartan were above the ULOQ in the case of six samples, which were subsequently diluted to determine the plasma concentration. It should be mentioned that this dilution step was not necessary to assess the adherence, as the ULOQ (125 ng/mL for olmesartan and torasemide and 250 ng/mL for valsartan) was within the calculated dose-dependent reference plasma concentration range in all cases.

In two cases containing bisoprolol (sample ID 13) or metoprolol (sample ID 7), the determined plasma concentrations were below the LLOQ, which was higher than c_min–20%_. Therefore, an adherence assessment was not possible in both cases. However, it must be mentioned that additional torasemide in the case of sample ID 13 was within the reference plasma concentration range. Furthermore, if the torasemide LLOQ would be elevated to the concentration of Cal 2 (25 ng/mL) due to the carryover, the adherence of the patients with sample IDs 11 and 18 to torasemide could not be assessed anymore.

Of course, the current procedure has limitations. First of all, calculating the dose-dependent reference plasma concentration ranges is time-consuming, but as soon as they are available, they can be used for patients receiving the same daily dose. Furthermore, most data from Baselt described plasma concentrations after a single dose [33], and higher plasma concentrations may be expected in a steady state. Interindividual differences, for example, in the expression of metabolic enzymes were not considered. Finally, some analytes were only contained in a single plasma sample, as only a limited number of samples was analyzed. As a future perspective, more plasma samples should be analyzed to further evaluate and improve the current dose-dependent reference plasma concentration ranges approach.

In summary, the measured plasma concentrations of 12 out of 19 samples were within the calculated reference plasma concentration ranges for all analyzed antihypertensive drugs (65%), of two times above (10%), and of three times below (15%). The adherence of sample IDs 7 and 13 could not be assessed, due to bisoprolol or metoprolol plasma concentrations and c_min–20%_ below the LLOQ. Fourteen out of 19 samples were classified as adherent (75%) and three as nonadherent (IDs 1, 4, and 10).

## 4. Materials and Methods

### 4.1. Chemicals, Reagents, and Human Biosamples

Amlodipine besylate was purchased from Pfizer (Karlsruhe, Germany); canrenone from the European Directorate for the Quality of Medicines & HealthCare (Strasbourg, France); bisoprolol fumarate, metoprolol tartrate, carvedilol, candesartan, valsartan, olmesartan, torasemide, and a diazepam-d5 solution (1 mg/mL in methanol) from LGC Standards (Wesel, Germany); atorvastatin-d5 calcium salt from Alsachim (Shimadzu Group, Illkirch Graffenstaden, France); and methanol, acetonitrile (ACN), diethyl ether, ethyl acetate, and natrium sulfate from VWR International GmbH (Darmstadt, Germany), as well as ammonium formate and formic acid from Merck KGaA (Darmstadt, Germany). All chemicals were of analytical grade. Water was purified with a Millipore filtration unit (18.2 Ω × cm water resistance) from Merck (Darmstadt, Germany). Drug-free pooled human blank plasma was obtained from a local blood bank. The analysis of human samples was approved by the local ethics committee to assess medication adherence using toxicological analyses objectively and complied with the Declaration of Helsinki. All patients provided written informed consent.

### 4.2. Preparation of Stock Solutions for Calibrators and Quality Controls

Stock solutions (1 mg/mL) of the antihypertensive drugs and of the IS atorvastatin-d5 were prepared in methanol. All solutions were stored at −20 °C. Methanolic mix solutions for the calibrators and QC samples were prepared using different stock solutions and freshly spiked in pooled human blank plasma during sample preparation. Amlodipine-containing solutions were handled in amber-colored vials during method development and validation. Final plasma concentrations of the calibrators (Cal 1−6), as well as QC samples (at four levels: LLOQ, low, medium, and high), are given in Table 1.

### 4.3. Sample Preparation

Plasma samples were prepared by LLE. First, 100 µL of blood plasma, 10 µL of formic acid (100%), 10 µL of diazepam-d5 (100 ng/mL in methanol), and 10 µL of methanol were mixed and vortexed for 10 s. For preparation of the calibrator and QC samples, methanol was replaced by 10 µL of the respective mix solution (concentrations given in Table 1). Afterwards, 100 µL of a saturated sodium sulfate solution were added, the mixture was vortexed for 10 s, and 500 µL of diethyl ether-ethyl acetate (1:1, *v/v*) were added. After another vortexing step (10 s), manual shaking for 30 s, and centrifugation (5 min, 18,407× *g*), 300 µL of the clear organic phase were transferred to an amber-colored vial and evaporated to dryness at 30 °C under a stream of nitrogen. The residue was reconstituted in 40 µL of a mixture of eluents A and B (60:40, *v/v*, 2-mM aqueous ammonium formate containing 0.1% formic acid and 1% ACN:ACN containing 2-mM ammonium formate, 0.1% formic acid, and 1% water). Reproducibility of the sample preparation procedure was tested by determination of the CVs of the peak area ratios of the analyte (*m/z* of M + H^+^, given in Table 2) and the IS diazepam-d5 at a plasma concentration of 100 ng/mL, each, after three extractions (*n* = 3).

### 4.4. UHPLC-ITMS Conditions

A Dionex UltiMate 3000 LC-system (Thermo Fisher Scientific, TF, Dreieich, Germany) connected to an amaZon speed ion trap mass spectrometer (Bruker Daltonik, Bremen, Germany) with an electrospray ionization source were used. Gradient elution was performed on a TF Acclaim RSLC120 C18 column (100 mm × 2.1 mm × 2.2 µm) at a column temperature of 45 °C using eluent A (2-mM aqueous ammonium formate containing 0.1% formic acid and 1% ACN) and eluent B (ACN containing 2-mM ammonium formate, 0.1% formic acid, and 1% water). The gradient was programmed as follows: 0 to 1 min hold 1% B, 1–4 min increase to 21% B, 4–10 min increase to 33% B, 10–13 min increase to 99% B, 13–15 min hold 99% B, 15 to 15.1 min decrease to 1% B, and 15.1–16 min hold 1% B, each step at a constant flow rate of 0.7 mL/min. The injection volume was 10 µL. The mass spectrometer operated in smartMRM mode using a scheduled precursor list (SPL) to generate MS^2^ spectra of scheduled precursors and a mass range from *m/z* 70–500 at *m/z/s* 32,500. SmartMRM mode parameters were programmed as follows: ionization mode, positive; ICC target (determines how many ions should be transferred into the trap), 50,000; max. accumulation time, 50 ms; MS spectra, interval 0.01; averages (determines how many scans will be summed and averaged for one spectrum), 1; SPS for tandem mass spectrometry (MS/MS) acquisition enabled; fast chromatography, not selected; edit parameters, link edit-enabled; and rolling averaging (filtering out short-term fluctuations), No. 1. Source parameters were as follows: capillary voltage, 4500 V; end plate offset, 500 V; nebulizer gas, 80 psi; dry gas, 10 L/min; and dry temperature, 159 °C.

### 4.5. Method Validation

Method validation was performed according to the “Guideline on bioanalytical method validation” of the EMA [30]. Drug identification was based on three criteria: first, the mass of the protonated precursor ion, second, the MS^2^ spectrum, and third, the t_R_. Data handling was done using Bruker Compass Data Analysis Version 4.4 (Bruker Daltonik). For quantification, peak area ratios of the analyte (*m/z* of M + H^+^ are given in Table 2) and the IS diazepam-d5 (M + H^+^ at *m/z* 290.11, t_R_ 11.30 min) were used. Graphs and structures of analytes were edited using CorelDraw X7 Version 17.0.0.491 (Munich, Germany). Statistical analysis was done using Microsoft Excel 2010 (Redmond, WA, USA) and GraphPad Prism 5.00 (GraphPad Software, La Jolla, CA, USA).

#### 4.5.1. Ion Suppression and Enhancement of Coeluting Analytes

Coelution and subsequent ion suppression and enhancement of possible IS was tested for diazepam-d5 (M + H^+^ at *m/z* 290.11) and diazepam (M + H^+^ at *m/z* 285.08), as well as atorvastatin-d5 (M + H^+^ at *m/z* 564.29) and atorvastatin (M + H^+^ at *m/z* 559.26). Substances in separate methanolic solutions (1 mg/L) were injected onto the UHPLC-ITMS system (*n* = 3). The absolute peak areas were compared to the absolute peak areas of a mixture (*n* = 3) containing both substances (1 mg/L), respectively.

#### 4.5.2. Selectivity

Ten plasma samples from different donors were extracted without the addition of IS diazepam-d5, individually analyzed, and evaluated for interferences in order to evaluate whether the current method can differentiate the analytes of interest and IS from endogenous components in the sample. The plasma samples also contained exogenous compounds such as ethanol, sedatives (e.g., midazolam), antidepressants (e.g., mirtazapine), antiepileptics (e.g., valproic acid), analgesics (e.g., metamizole), other antihypertensives (e.g., urapidil), or local anesthetics (e.g., mepivacaine), but none of the cardiovascular drugs were included in the current method development. Selectivity was given if the response was less than 20% of the LLOQ for the antihypertensive drugs and less than 5% for the IS diazepam-d5 [30].

#### 4.5.3. Carryover

For carryover testing, an extract of pooled blank plasma was injected after the highest calibration standard (Cal 6, plasma concentrations of analytes; see Table 1). Carryover should not be greater than 20% of the LLOQ for the antihypertensive drugs and 5% for IS diazepam-d5 [30].

#### 4.5.4. Calibration

Six calibrator (Cal 1–6) and four QC (LLOQ, low, medium, and high) levels were prepared for each analyte by spiking pooled human blank plasma with predefined analyte concentrations, which are given in Table 1. The LLOQ was defined to be equal to the concentration of Cal 1 and the upper limit of quantification (ULOQ) to the concentration of Cal 6. In total, four different calibration ranges with individual LLOQ and ULOQ levels were defined depending on the analyte. A blank sample (extracted sample without IS diazepam-d5 or analytes) and zero sample (extracted sample with IS diazepam-d5 but without analytes) were prepared. QCs were prepared in duplicates (*n* = 2), and each calibrator, QC, blank, and zero sample was injected and analyzed once. For evaluation, linear and nonlinear regression models were tested, as well as different weighing factors (equal, 1/x, and 1/x^2^). The AC for the back-calculated concentrations of the calibrator standards were set to ±15% of the nominal value, except for the LLOQ, for which the back-calculated concentration should be within ±20% [30]. At least 75% of the calibration standards must fulfill these criteria, meaning that, in the current case, five out of six calibrator samples must fulfill the criteria.

#### 4.5.5. Matrix Effect

According to the EMA guidelines, single plasma samples from six different donors were used instead of the pooled matrix, and two different sample sets were prepared with a low and a high concentration each (for concentrations of low and high QC, see Table 1). The first sample set contained analytes in a pure methanolic solution (neat standard). For the second sample set, blank plasma was extracted and spiked with analytes afterwards.

For each analyte and the IS, the MF was calculated for each lot of the matrix as the ratio of the peak area in the presence of the matrix (sample set 2) and the peak area in the absence of the matrix (sample set 1). Finally, the IS-normalized MF was calculated by dividing the MF of the analyte by the MF of the IS. The CV of the IS-normalized MF calculated from the 6 lots of the matrix should not be greater than 15%.

#### 4.5.6. Accuracy and Precision

The closeness of the determined value obtained by the method to the nominal concentration of the analyte is described by the accuracy, which is expressed in percentage. The closeness of repeated individual measures of the analyte is described by the precision that can be expressed as the CV [30]. Freshly prepared QC samples were analyzed against a freshly prepared calibration.

##### Within-Run Accuracy and Precision

Within-run accuracy and precision were determined by analyzing five replicates of QCs (LLOQ and low, medium, and high QC; concentration levels given in Table 1) in a single run. The mean concentration should be within ±15% of the nominal concentration, except for the LLOQ, where it should be within ±20% of the nominal value, according to the EMA [30]. For within-run precision, CVs were calculated and should be within 15%, except for the LLOQ, where it should be within 20%.

##### Between-Run Accuracy and Precision

Between-run accuracy and precision were determined by analyzing five replicates of QCs (LLOQ and low, medium, and high QC; concentration levels given in Table 1) over three runs on three different days. The mean concentration should be within ±15% of the nominal concentration, except for the LLOQ, where it should be within ±20% of the nominal value, according to the EMA [30]. For between-run precision, CVs were calculated and should not be >15%, except for the LLOQ, where it should not be >20%.

##### Dilution Integrity

Dilution integrity was tested by spiking pooled human blank plasma with an analyte concentration five times higher than the high QC (for concentrations, see Table 1). After 1:10 dilution with pooled blank plasma, concentrations of medium QC were achieved (concentrations given in Table 1). Samples were extracted and analyzed as described above. Five replicates were prepared (*n* = 5). Accuracy and precision should be within 15%, according to the EMA [30].

#### 4.5.7. Stability of Stock Solutions

A methanolic mixture prepared from the stock solutions of the calibrators containing the antihypertensive drugs and the IS diazepam-d5 (1 mg/L, each) was analyzed once a week over a period of ten weeks, and CVs of the peak area ratios of the analyte and the IS were calculated. Between the analyses, the mixture was stored at −20 °C in an amber-colored vial. CVs not greater than 15% were defined as acceptable. Ten washing runs injecting an eluent mixture A/B (1:1, *v/v*) immediately after analysis prevented carryover.

#### 4.5.8. Autosampler Stability

Plasma samples were extracted and analyzed immediately after preparation (t0) and after the appropriate storage condition (t1) against a freshly spiked calibration. Stability of the processed samples (low and high QC; concentrations given in Table 1, *n* = 3, respectively) in the autosampler was evaluated by reanalyzing the processed samples stored for 20 h in the autosampler (8 °C). The mean concentration at each concentration level should be within ±15% of the nominal concentration.

### 4.6. Applicability and Calculation of the Dose-Dependent Reference Plasma Concentration Range

A total of 19 plasma samples of hypertensive patients were extracted and analyzed, as described above. The lower limit of the reference plasma concentration range was represented by c_min–20%_ (=calculated plasma concentration 24 h after the latest drug intake minus 20%) and the upper limit by the peak plasma concentration c_max_. The latter was taken from Baselt [33], as well as the corresponding time when the c_max_ was reached, t_max_, and the elimination half-life, t_1/2_. The shortest given elimination half-life t_1/2_ was always used for the calculation of the trough concentration c_min_. A summary of the pharmacokinetic parameters used for calculation of the reference plasma concentration ranges, as well as an example for such a calculation, are available as Supplementary Materials (Tables S2 and S3, respectively). If the measured plasma concentration was above the ULOQ of the corresponding analyte, the sample was diluted, extracted, and reanalyzed.

## 5. Conclusions

An analytical procedure for the simultaneous quantification of nine cardiovascular drugs based on the LLE of only 100 µL of blood plasma and fast UHPLC separation within 12 min, followed by an ITMS analysis, was successfully developed. Validation was successfully performed in accordance with the international guidelines. Limitations exceeded the AC for the matrix effects in the case of amlodipine and its instability. The developed procedure was used for the analysis of 19 plasma samples containing a total of 28 antihypertensive drugs to determine whether a patient can be considered as adherent or not. For this purpose, the dose-dependent reference plasma concentration ranges were calculated using pharmacokinetic parameters. The adherence of two subjects out of 19 could not be assessed (10%), due to the measured plasma concentrations and c_min–20%_ below the LLOQ, but 14 were classified as adherent (75%) and three as nonadherent (15%). In contrast, all 19 (100%) were claimed adherent based on the results of the qualitative urine screening due to the presence of the antihypertensive drug and/or metabolites in the urine. The current pilot study demonstrated that the interpretation of plasma concentrations is far more sophisticated and time-consuming than urine screening results, and further data is needed to estimate whether plasma quantification is superior to urine screening in confirming the adherence to antihypertensive medication.

## Figures and Tables

**Table 1:** Final plasma concentrations (ng/mL) of the antihypertensive drugs in the six calibrator samples (Cal 1–6) and quality control samples (QC) at four levels (LLOQ, low, medium, and high), as well as weighting factors used in a linear calibration model. Lower limit of quantification (LLOQ), upper limit of quantification (ULOQ), and medium (Med).

Analyte	Cal 1 (≡LLOQ)	Cal 2	Cal 3	Cal 4	Cal 5	Cal 6 (=ULOQ)	LLOQ QC	Low QC	Med QC	High QC	Weigh-ting
Amlodipine	10	50	100	150	200	250	10	30	120	240	1/x
Bisoprolol	5	25	50	75	100	125	5	15	60	120	1/x^2^
Candesartan	10	50	100	150	200	250	10	30	120	240	1/x^2^
Canrenone	20	100	200	300	400	500	20	60	240	480	1/x^2^
Carvedilol	1	5	10	15	20	25	1	3	12	24	1/x^2^
Metoprolol	10	50	100	150	200	250	10	30	120	240	1/x^2^
Olmesartan	5	25	50	75	100	125	5	15	60	120	1/x
Torasemide	5	25	50	75	100	125	5	15	60	120	equal
Valsartan	10	50	100	150	200	250	10	30	120	240	1/x

**Table 2:** Precursor ion *m/z* after positive ionization (M + H^+^), retention order, and retention times (t_R_) of the antihypertensive drugs and internal standards (IS).

Analyte	Precursor Ion *m/z*	Retention Order	t_R_, min
Amlodipine	409.15	6	8.8
Bisoprolol	326.23	2	5.2
Candesartan	441.17	7	10.5
Canrenone	341.21	10	11.7
Carvedilol	407.20	5	7.8
Diazepam-d5 (IS)	290.11	8	11.3
Metoprolol	268.19	1	4.2
Olmesartan	447.21	3	5.7
Torasemide	349.13	4	6.1
Valsartan	436.23	9	11.5

**Table 3:** Internal standard (IS)-normalized matrix factors of the analytes and coefficients of variation (CV) derived from measurements of six lots of the matrix at a low (low QC, see Table 1) and a high concentration (high QC, see Table 1).

Analyte	Low QC	CV, %	High QC	CV, %
	IS-Normalized Matrix Factor		IS-Normalized Matrix Factor	
Amlodipine	2.54	26 *	1.37	16 *
Bisoprolol	1.12	11	1.07	13
Candesartan	0.82	24 *	0.96	17 *
Canrenone	1.10	11	0.99	10
Carvedilol	1.15	19 *	1.08	7
Metoprolol	1.16	17 *	1.07	10
Olmesartan	0.95	22 *	0.96	17 *
Torasemide	1.06	12	1.08	12
Valsartan	1.12	42 *	1.07	25 *

Values outside the recommended European Medicines Agency (EMA) guideline limits [30] are marked with an asterisk (*).

**Table 4:** Quantification results of 19 plasma samples containing 28 antihypertensive drugs in total, along with the prescribed daily doses, therapeutic plasma concentration ranges according to Schulz et al. [32], elimination half-lives t_1/2_, time after intake when the peak plasma concentration was reached (t_max_), and calculated dose-dependent reference plasma concentration ranges (c_min–20%_-c_max_). The dose used for calculation of the reference plasma concentration range was given in parentheses if it was different from the patient´s prescribed daily dose. Plasma concentrations below the LLOQ are indicated by <LLOQ. Plasma concentrations below the given concentration ranges are marked by (↓), within by (⟷), and above by (↑). If the measured plasma concentration cannot be classified using the given concentration ranges, they are marked by (?).

Analyte	Sample ID	Measured Plasma Conc. ng/mL	Daily Dose, mg	Therapeutic Plasma Conc., ng/mL [32]	t_1/2_, h [33]	t_max_, h [33]	Calculated Reference Plasma Conc., ng/mL
Bisoprolol	2	5.9	2.5	10–100 (↓)	7–15	2	2.0–21 (5 mg) (⟷)
	4	27	10	10–100 (⟷)			4.8–52 (⟷)
	6	17	5	10–100 (⟷)			2.0–21 (⟷)
	9	9	5	10–100 (↓)			2.0–21 (⟷)
	12	17	5	10–100 (⟷)			2.0–21 (⟷)
	13	<LLOQ (5 ng/mL)	2.5	10–100 (↓)			2.0–21 (5 mg) (?)
	14	5.5	5	10–100 (↓)			2.0–21 (⟷)
Candesartan	12	66	8	80–400 (↓)	8–13		9.2–61 (↑)
	14	160	32	80–400 (⟷)		4	39–260 (⟷)
	15	92	32	80–400 (⟷)			39–260 (⟷)
	16	83	16	80–400 (⟷)			18–120 (⟷)
	17	49	16	80–400 (↓)			18–120 (⟷)
Canrenone	4	90	50	50–500 (⟷)	13–23	2	160–600 (100 mg) (↓)
Carvedilol	18	2.4	6.25	20–300 (↓)	4–7	1	0.36–58 (12.5 mg) (⟷)
Metoprolol	5	38	50	20–600 (⟷)	2.5–7.5	1	0.10–72 (⟷)
	7	<LLOQ (10 ng/mL)	100	20–600 (↓)		2	0.26–140 (?)
Olmesartan	1	52	40	100–1000 (↓)	6–15	2	55–830 (↓)
	3	1100	40	100–1000 (↑)			55–830 (↑)
	10	14	20	100–1000 (↓)			26–390 (↓)
Torasemide	6	41	10	64–2000 (↓)	2–6	1	0.47–1600 (⟷)
	8	240	10	64–2000 (⟷)			0.47–1600 (⟷)
	11	18	10	64–2000 (↓)			0.47–1600 (⟷)
	13	30	10	64–2000 (↓)			0.47–1600 (⟷)
	14	170	5	64–2000 (⟷)			0.47–1600 (10 mg) (⟷)
	18	9.2	2.5	64–2000 (↓)			0.47–1600 (10 mg) (⟷)
Valsartan	6	920	320	800–6000 (⟷)	4–12	3	240–11,000 (⟷)
	11	3000	320	800–6000 (⟷)			240–11,000 (⟷)
	19	410	320	800–6000 (↓)			240–11,000 (⟷)

## Data Availability

The data can be made available upon reasonable request.

## Supplementary Materials

The following are available online: Table S1: Parameters of three different, freshly prepared calibrations acquired during validation procedure (slope, intercept, and deviation of back-calculated concentrations of calibrators. Table S2: Summary of the pharmacokinetic data (elimination half-live t_1/2_, peak plasma concentration cmax, and time when cmax was reached, tmax) used for calculating the dose-dependent reference plasma concentration ranges. Table S3: Example calculation of the lower limit (c_min–20%_) of the reference plasma concentration range for 10 mg bisoprolol daily.
